# Effects of a chickpea protein hydrolysate on exercise-induced biomarkers of muscle damage and inflammation in federated road cyclists

**DOI:** 10.1080/15502783.2026.2723237

**Published:** 2026-10-03

**Authors:** Jesús García-Durán, Antonio J. Sánchez-Oliver, Noelia M. Rodríguez-Martín, María-Soledad Fernández-Pachón, Justo Pedroche, Francisco Millán, José Antonio González-Jurado

**Affiliations:** a Faculty of Sports Science, Pablo de Olavide University, Seville, Spain; b Departamento de Motricidad Humana y Rendimiento Deportivo, Universidad de Sevilla, Seville, Spain; c Group of Plant Proteins, Instituto de la Grasa-CSIC, Seville, Spain; d Area of Nutrition and Food Sciences, Department of Molecular Biology and Biochemistry Engineering, Universidad Pablo de Olavide, Seville, Spain

**Keywords:** Recovery, endurance athletes, cycling, plant-based protein, chickpea protein

## Abstract

**Background:**

This randomized, double-blind, placebo-controlled trial examined the effects of a chickpea protein hydrolysate (CPH) on biomarkers associated with exercise-induced muscle damage and inflammation in federated male road cyclists.

**Methods:**

Twenty-four participants completed a 14-day intervention receiving either 12 g/day of chickpea protein hydrolysate (EXP) or a placebo (PLA) following training sessions. The selected dose was based on previous studies demonstrating antioxidant and immunomodulatory bioactive properties of the hydrolysate. Nutritional intake and training were registered. Body composition and biochemical markers of muscle damage and inflammation were assessed before and after the study.

**Results:**

No significant differences were observed in body composition, training load, or dietary intake between groups, indicating controlled experimental conditions. However, between-group analyses of change scores revealed significant differences for lactate dehydrogenase (LDH; *p* = 0.04) and C-reactive protein (CRP; *p* = 0.02), favoring the experimental group. LDH decreased in the EXP group while increasing slightly in the placebo group, whereas CRP decreased in EXP and increased in PLA. Although creatine kinase (CK) showed a favorable pattern in the EXP group, the between-group comparison did not reach statistical significance (*p* = 0.062). Myoglobin followed a similar downward trend. The high baseline protein intake (>2 g/kg/day) and short intervention duration may have limited changes in body composition, nevertheless, the observed biomarker responses suggest that CPH supplementation may influence selected physiological processes associated with exercise-induced inflammation and cellular stress.

**Conclusions:**

In trained cyclists with high habitual protein intakes, 14 days of CPH supplementation did not alter body composition but was associated with favorable changes in selected biomarkers related to exercise-induced inflammation and cellular stress compared with placebo. Given the short intervention period and the absence of direct assessments of recovery or performance, these findings should be interpreted as short-term biochemical responses. Further studies are required to determine their physiological relevance and whether these biomarker changes translate into meaningful improvements in recovery, training adaptation, or athletic performance.

## 1. Introduction

Protein is a fundamental nutrient for exercise recovery and adaptation, providing the amino acid substrates necessary for muscle repair and remodelling after physical stress. It can be obtained from both animal and plant sources or as supplements derived from them [1]. The nutritional quality of a protein source depends primarily on its amino acid composition and digestibility, which together determine its capacity to support muscle protein synthesis (MPS) and recovery [2]. High-quality proteins supply adequate quantities of all essential amino acids, particularly leucine, and are efficiently digested and absorbed to stimulate anabolic signalling pathways [1]. Numerous studies have shown that protein ingestion in proximity to exercise reduces markers of muscle damage, attenuates performance decrements, and accelerates recovery [3–6]. When consumed before and immediately after exercise, protein has been shown to increase mRNA expression of anabolic regulators and prevent post-exercise declines in myogenic markers [5], thereby promoting faster recovery and tissue remodelling.

Although most research on protein supplementation has focused on resistance-trained individuals, endurance athletes may also benefit from increased protein availability, especially during periods of high training loads and elevated energy demands [7]. Protein supplementation in endurance athletes has been associated with attenuated muscle damage, improved remodelling of skeletal muscle tissue, and enhanced tolerance to training stress [8–11]. However, the literature is not unanimous; several studies have reported no significant improvements in performance or recovery when protein is added during periods of heavy endurance training [12–14]. Nonetheless, the consistent mechanistic evidence linking protein intake to enhanced MPS and reduced muscle damage underlines its importance in recovery-oriented sports nutrition [1].

Protein supplements rank among the most commonly consumed nutritional products worldwide, with their market value expected to reach approximately USD 21.5 billion by 2025. Within this context, sports nutrition represents their primary area of application, and plant-based proteins constitute the most rapidly expanding segment of this industry [15]. Whey protein, a by-product of cheese production, remains the most widely consumed and studied supplement due to its high biological value, rapid digestibility, and favourable amino acid profile rich in branched-chain amino acids (BCAA) [16,17]. Numerous investigations have demonstrated the ergogenic effects of whey protein on muscle recovery, strength development, and body composition [18,19]. Beyond its anabolic effects, whey exhibits additional bioactive properties, including antioxidant, cardioprotective, anti-obesity, and antidiabetic activities [20]. However, the growing global population, finite natural resources, and increasing environmental awareness have stimulated interest in alternative, more sustainable protein sources to meet future nutritional needs [21].

In recent years, plant-based proteins have gained prominence as viable alternatives to animal-derived supplements, driven by environmental, ethical, and health considerations [17,22]. Plant proteins are generally characterised by lower digestibility and incomplete amino acid profiles, particularly limited in lysine, methionine, or leucine, which may reduce their anabolic potential compared to animal proteins [16,23,24]. Nevertheless, advances in food technology have led to the development of plant protein isolates, blends, and hydrolysates with improved nutritional and functional characteristics. For example, combinations such as pea–rice or canola–potato can match whey’s efficacy when consumed in appropriate doses (≥30 g providing ~2.5 g leucine) [25–27]. These developments have contributed to a paradigm shift in sports nutrition, emphasising sustainability without compromising performance outcomes.

Beyond their amino acid contribution, plant proteins and their hydrolysates contain bioactive compounds that may confer additional physiological benefits. Several studies indicate that plant-rich diets reduce oxidative stress and systemic inflammation [28,29]. Oxidative stress arises from an imbalance between reactive oxygen species (ROS) production and antioxidant defenses, leading to cellular damage and impaired recovery [30–32]. Exercise, particularly of high intensity or long duration, transiently increases ROS generation, which can activate proinflammatory cytokines, impair muscle protein synthesis, and contribute to chronic fatigue or overtraining if unmitigated [33]. In this context, antioxidant and anti-inflammatory strategies—whether through polyphenol-rich foods or plant protein supplementation—may play a valuable role in recovery and adaptation [34–36]. Plant proteins such as soy and oat, which contain bioactive components like *β*-conglycinin, avenanthramides, and *β*-glucans, have demonstrated beneficial effects on glucose regulation, inflammation, and oxidative balance [36–39]. These properties support the rationale for exploring plant proteins not only as anabolic substrates but also as functional compounds with recovery-enhancing potential.

Among the available plant sources, legumes have drawn considerable attention due to their favourable nutritional composition, high protein content, and presence of peptides with antioxidant and anti-inflammatory activities [40–42]. In particular, chickpea (Cicer arietinum) is an underutilized but promising source, providing a balanced amino acid profile, complex carbohydrates, dietary fibre, and a wide range of bioactive molecules [42]. Previous studies conducted with the same chickpea protein hydrolysate (CPH) used in the present investigation demonstrated a high content of low-molecular-weight bioactive peptides (<2.88 kDa), high digestibility, excellent solubility, and marked antioxidant activity measured through trolox equivalent antioxidant capacity (TEAC), ferric reducing antioxidant power (FRAP), and oxygen radical absorbance capacity (ORAC) assays. Furthermore, cellular studies showed its ability to modulate inflammatory signalling pathways, including nuclear factor kappa B (NF-κB) and NOD-like receptor family pyrin domain-containing 3 (NLRP3)-related responses. These characteristics provided the biological rationale for evaluating whether the hydrolysate could influence biomarkers associated with exercise-induced muscle damage and inflammation in trained athletes [43,44].

Despite the growing body of literature on plant-based proteins, evidence regarding the effects of CPH on post-exercise recovery in athletes remains scarce [7,45]. Given the physiological demands of endurance sports such as road cycling—characterised by prolonged exercise bouts, frequent intensity fluctuations, and substantial oxidative stress [46,47]—developing sustainable nutritional strategies to enhance recovery is of particular relevance [48–50]. Therefore, the present study aimed to analyse the effects of CPH supplementation on biomarkers of muscle damage and inflammation and body composition in male road cyclists. By providing new insights into the efficacy of a novel plant-based protein hydrolysate, this research contributes to the scientific understanding of sustainable sports nutrition and its role in optimising recovery in high-demand endurance contexts.

## 2. Materials and methods

### 2.1. Experimental design

This study was structured as a randomised, double-blind, placebo-controlled trial intervention. The total duration was 14 days, during which participants were assigned to two randomised groups: experimental (EXP) and placebo (PLA), each consisting of 12 cyclists. The allocation of the two supplements (placebo/protein hydrolysate) was coded by personnel external to the research team, ensuring neutrality in administration. The supplements were consumed in single-dose format immediately after each training session and at the same time on rest days.

### 2.2. Sample

Twenty-four male road cyclists residing in the province of Seville (Spain) participated in the study, all holding a valid federation license from the Andalusian Cycling Federation (FAC). Initially, 40 volunteers were recruited, of whom 25 met the inclusion criteria. Ultimately, 24 completed the study, with one dropout due to reasons unrelated to the intervention.

The inclusion criteria were: no consumption of nutritional supplements or anti-inflammatory/antioxidant drugs in the 30 days prior; no legume allergies; federation membership during the previous year; consistent training; no recent participation in similar studies; commitment to protocol adherence (≥95%); and full understanding of the informed consent. Exclusion criteria included failure to meet the above requirements, lack of cooperation, health risks, or metabolic disorders. The following table presents the descriptive data of the sample (Table 1).

**Table 1.** Age, height and body mass of the sample, placebo and experimental group.

Group	Age (years)	Height (cm)	Body Mass (kg)
ALL (*n* = 24)	42.9 ± 6.4	179.3 ± 6.8	78.1 ± 2.4
PLA (*n* = 12)	42.0 ± 7.2	179.0 ± 7.4	80.5 ± 12.2
EXP (*n* = 12)	43.8 ± 5.7	179.0 ± 6.6	75.7 ± 8.8

### 2.3. Supplementation

The active supplement consisted of a chickpea protein hydrolysate (CPH) developed by the Plant Protein Group of the Institute of Fat (Instituto de la Grasa) at the Spanish National Research Council (CSIC; https://www.ig.csic.es/es/), following the methodology described by Rodríguez-Martín et al. (2024) [44]. The final formulation and packaging of the supplement were carried out by Aurora Intelligent Nutrition S.L. The formulation of the supplement is protected under an industrial patent owned by Aurora Intelligent Nutrition S.L.

Both supplements were provided as powdered formulations in identical coded sachets and dissolved in 550 mL of water prior to consumption. The composition of the experimental and placebo supplements is presented in Table 2. The experimental supplement contained chickpea protein hydrolysate (H30BIO) as the main ingredient, whereas the placebo formulation contained maltodextrin in its place. Both products contained the same excipients, including vitamin C, acidity regulators, sweeteners, and natural flavourings. Product coding and allocation were performed by personnel external to the research team, and both participants and investigators remained blinded to treatment assignment throughout the study.

Supplements were consumed once daily immediately following training sessions and at the same time of day on rest days. Although the formulations were designed to be visually similar, complete taste matching could not be guaranteed due to the intrinsic sensory characteristics of the protein hydrolysate. The supplements were not strictly isocaloric; however, the small difference in energy content was considered unlikely to influence the measured outcomes.

**Table 2.** Composition of experimental and placebo supplements.

Component	PLA (%)	EXP (%)
Chickpea protein hydrolysate (H30BIO)	0	84
Maltodextrin	93	9
Excipients*	7	7

^*^ Excipients included vitamin C, acidity regulators, sweeteners, and natural flavourings.

The 12 g/day dose of chickpea protein hydrolysate was selected based on previous physicochemical and biological characterisation studies of the same hydrolysate [43,44], which demonstrated that this hydrolysate contains bioactive peptides with antioxidant and immunomodulatory potential. Given that the participants already reported a high habitual protein intake (>2 g·kg^−1^·day^−1^), the purpose of the intervention was not to substantially increase total protein intake or maximise muscle protein synthesis, but rather to evaluate whether a practical and realistic daily dose of CPH could modulate biomarkers associated with exercise-induced muscle damage and inflammation. Furthermore, the selected dose was considered appropriate for daily use by athletes and representative of a feasible supplementation strategy under real-world conditions.

### 2.4. Data custody and protection

Data and biological samples were stored at the CIRFD (Physical Performance & Sports Research Centre) of Pablo de Olavide University, with restricted access to the research team. The study was approved by the Andalusian Biomedical Research Ethics Portal (PEIBA), internal code: 2824-*N*-21, and all participants signed informed consent in accordance with Organic Law 3/2018 on Personal Data Protection. Ethical principles of the Declaration of Helsinki and international bioethics agreements were respected. This study was retrospectively registered at ClinicalTrials.gov (Identifier: PID2019-111368RB-100 - provisional registration identifier).

### 2.5. Data collection

Variables related to training, nutrition, body composition, and blood biomarkers were recorded.

#### 2.5.1. Training data

Cyclists recorded their sessions using the Strava© and Garmin Connect© applications. The weekly training plan included four cycling sessions, two strength sessions, and one rest day. Intensity was monitored using heart rate (HR) and the rating of perceived exertion (RPE) scale. HR zones were individually estimated, prioritising work in zones II and III, except on the day prior to blood sampling, when a high-intensity session (zone V) was performed [51]. The high-intensity cycling session performed on the day prior to blood sampling lasted 120 min and included a 25-30 min warm-up, 10 × 1 km intervals at 90-95% of maximal heart rate (HRmax) with 1 min recovery periods, and low-intensity cycling until session completion.

Strength training was conducted twice per week, following a muscular endurance protocol: five sets of 20 repetitions per exercise, with a load of 50–60% of one-repetition maximum (1RM), slow execution speed, and 1.5 minutes of rest between sets. Exercises included: squats, lat pulldown, leg press, chest press, barbell row, leg curl and dumbbell row.

#### 2.5.2. Nutritional data

Nutritional intake over six days was recorded using 24-hour dietary recalls via the FatSecret app, three days per week: one rest day, one cycling training day, and one strength training day. Macronutrients (carbohydrates, proteins, and fats) were analysed, and prior nutritional guidelines were provided to homogenise the data. These guidelines were based on a balanced diet, adjusted to individual energy demands, prioritising minimally processed foods and high-quality sources for each macronutrient.

#### 2.5.3. Anthropometric measurements

Body composition was assessed using segmental bioelectrical impedance analysis (Tanita BC-418 MA), recording body mass, fat mass, and lean mass. Height was measured with a digital stadiometer (AD-6227), and body mass index (BMI) was calculated. Basal metabolism was estimated automatically by the Tanita BC-418 MA using the manufacturer’s proprietary predictive algorithm derived from the anthropometric and body composition variables measured during the assessment.

#### 2.5.4. Blood samples

Blood samples were collected in a fasting state, following a high-intensity cycling session and 12 hours of rest. Biochemical and haematological parameters were analysed, including glucose, insulin, creatine kinase (CK), myoglobin, C-reactive protein (CRP), and lactate dehydrogenase (LDH).

### 2.6. Statistical analysis

Descriptive analysis included the calculation of mean and standard deviation as measures of central tendency and dispersion, respectively. For reliability analysis, 95% confidence intervals of the means were calculated. Before inferential analyses, the assumptions of normality and homoscedasticity were assessed using the Shapiro-Wilk and Levene’s tests, respectively. Within-group comparisons of pretest vs posttest changes were performed using either paired-samples t-tests or Wilcoxon signed-rank tests, depending on the normality. Between-group comparisons were conducted using a Generalised Linear Model (GLM), with baseline (pre-test) values included as a covariate when the assumptions of normality and/or homoscedasticity were not met. Effect sizes were calculated using Cohen’s d. Statistical significance was set at *p* < 0.05. All analyses were performed using IBM SPSS Statistics® version 29.

## 3. Results

### 3.1. Body composition and basal metabolism

Table 3 presents body composition and basal metabolism data before and after the intervention period for the total sample, the PLA group, and the EXP group. No statistically significant pre-post differences were found in any variable across any group.

**Table 3.** Pre and post-intervention data on body composition and basal metabolism.

	ALL (*n* = 24)		PLA (*n* = 12)		EXP (*n* = 12)	
	Pre	Post	Pre	Post	Pre	Post
Total Mass (kg)	78.1 ± 10.7	77.6 ± 10.5	80.5 ± 12.2	79.6 ± 11.9	75.7 ± 8.8	75.5 ± 9.0
BMI (kg/m^2^)	24.2 ± 2.2	24.1 ± 1.9	24.9 ± 2.5	24.6 ± 2.3	23.4 ± 1.6	23.6 ± 1.5
BM (kcal)	1852.8 ± 199.1	1853.5 ± 199.7	1900.8 ± 233.5	1900.8 ± 233.5	1804.7 ± 152.7	1806.1 ± 154.7
Fat Mass (%)	13.5 ± 4	13.6 ± 3.9	14.9 ± 4.2	15.2 ± 4.3	12.2 ± 3.6	12.1 ± 2.9
Fat Mass (kg)	10.8 ± 4.5	10.9 ± 4.4	12.3 ± 5.0	12.5 ± 5.1	9.3 ± 3.6	9.3 ± 3.0
Lean Mass (kg)	67.3 ± 7.1	66.7 ± 6.9	68.2 ± 8.1	67.2 ± 7.4	66.5 ± 6.1	66.2 ± 6.5

Values are presented as mean ± standard deviation. BMI: Body Mass Index; BM: Basal Metabolism.

### 3.2. Cycling and strength training sessions

Detailed data on cycling and resistance training sessions for the total sample, the placebo group, and the EXP group are presented in Tables 4 and 5, respectively. No statistically significant differences were found between the PLA and EXP groups across any of the cycling-related variables. Likewise, no statistically significant differences were found between the PLA and EXP groups for any of the resistance training session variables. This indicates that, within the limits of the available data and variability, both groups performed similarly across all measured metrics.

**Table 4.** Cycling training data.

	ALL (*n* = 24)	PLA (*n* = 12)	EXP (*n* = 12)
Average Km/day	72.3 ± 6.6	72.5 ± 7.3	72.1 ± 6.2
Total Km/Week 1º	250.1 ± 22.0	262.6 ± 20.9	237.7 ± 15.3
Total Km/Week 2º	243.3 ± 16.4	247.1 ± 21.6	239.5 ± 7.8
Average Weekly Km	246.7 ± 14.7	254.8 ± 15.5	238.6 ± 8.2
Total Km/14 Days	493.4 ± 27.3	509.7 ± 30.9	477.2 ± 23.1
Total Hours/Week 1º	8.6 ± 0.7	8.6 ± 0.7	8.7 ± 0.7
Total Hours/Week 2º	8.5 ± 0.7	8.3 ± 0.8	8.7 ± 0.6
Total Cycling Hours/14 Days	17.2 ± 1.3	17.0 ± 1.3	17.4 ± 1.2
Average Total Cycling Sessions	7.9 ± 0.3	7.9 ± 0.3	7.9 ± 0.3

Values are presented as mean ± standard deviation.

**Table 5.** Resistance training session data.

	Total (*n* = 24)	PLA (*n* = 12)	EXP (*n* = 12)
Number of Sessions	48	48	48
Total Training Time (min)	604	600	607
Session Duration (min)	50 ± 6	50 ± 7	51 ± 5
Maximum Repetition (% of 1RM)	59.0 ± 3.5	60.0 ± 1.4	58.0 ± 4.5
RPE (0–10)	7.3 ± 0.6	7.2 ± 0.7	7.5 ± 0.5

Values are presented as mean ± standard deviation.

### 3.3. Nutritional information recording

Nutritional intake was recorded by the cyclists using 24-hour dietary recalls on three different days of the week, covering one rest day, one cycling training day, and one strength training day. Table 6 details the recorded data for the total sample, the placebo group, and the EXP group. Figure 1 displays the reported intake data for total calories and each macronutrient across the studied groups and the total sample.

**Table 6.** Nutritional intake data.

	ALL (*n* = 24)	PLA (*n* = 12)	EXP (*n* = 12)
Calories—Rest Day	2022 ± 171	1961 ± 182	2043 ± 152
Calories—Cycling Session	3003 ± 353	3011 ± 295	2995 ± 409
Calories—Strength Session	2721 ± 310	2792 ± 246	2650 ± 354
Average Daily Calories	2582 ± 278	2588 ± 240	2653 ± 305
Carbohydrates—Rest Day (g)	269 ± 59	255 ± 71	282 ± 42
Carbohydrates—Cycling Session (g)	330 ± 49	325 ± 47	336 ± 51
Carbohydrates—Strength Session (g)	294 ± 38	297 ± 36	290 ± 41
Average Daily Carbohydrates (g)	298 ± 49	292 ± 51	303 ± 47
Protein—Rest Day (g)	143 ± 31	139 ± 35	147 ± 27
Protein—Cycling Session (g)	165 ± 23	160 ± 23	170 ± 22
Protein—Strength Session (g)	145 ± 23	146 ± 23	144 ± 24
Average Daily Protein (g)	151 ± 26	148 ± 27	154 ± 25
Fat—Rest Day (g)	73 ± 17	74 ± 21	71 ± 11
Fat—Cycling Session (g)	83 ± 12	83 ± 13	83 ± 10
Fat—Strength Session (g)	78 ± 13	78 ± 14	77 ± 13
Average Daily Fat (g)	78 ± 14	78 ± 17	77 ± 11

Values are presented as mean ± standard deviation.

**Figure 1.**
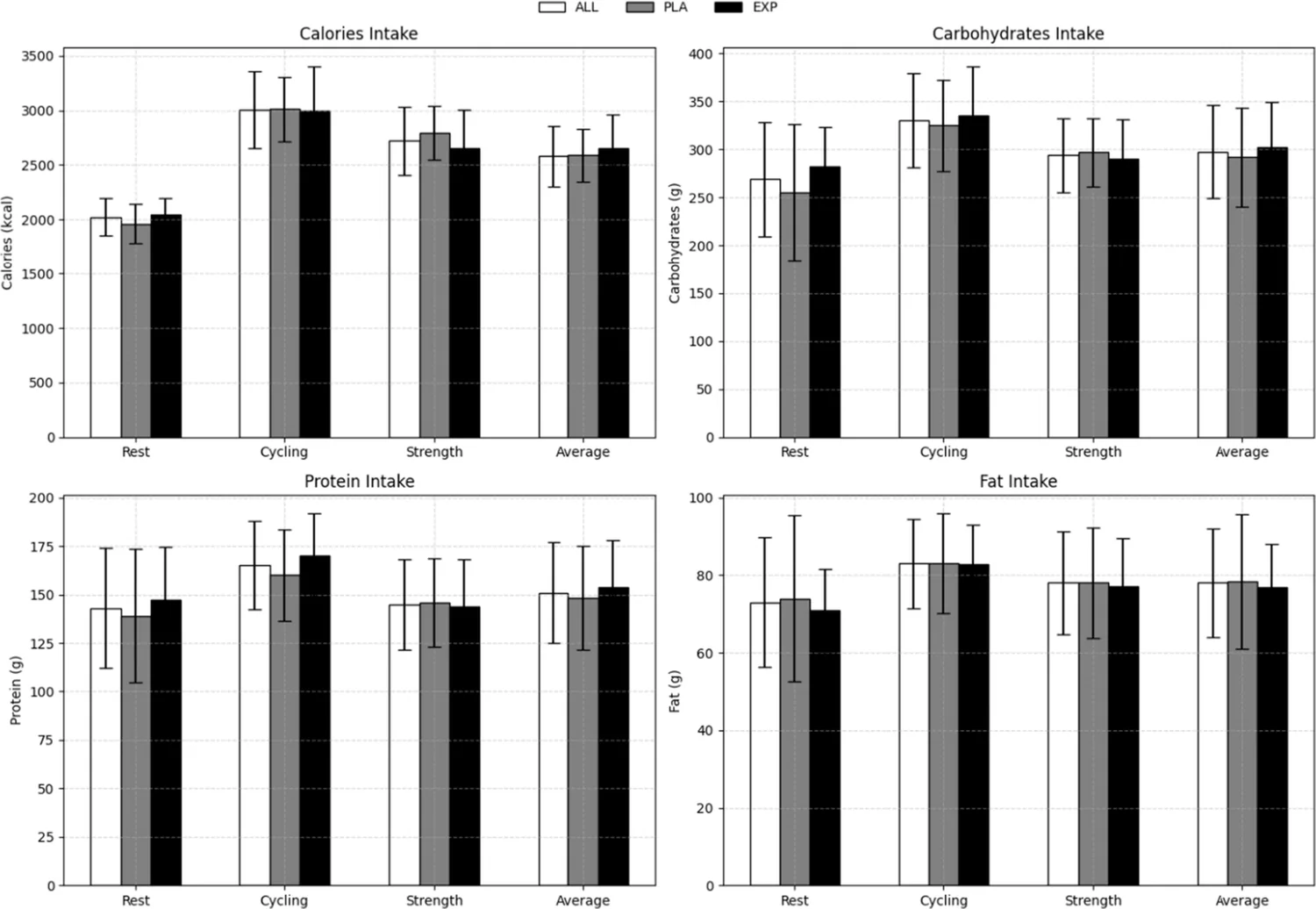
Average distribution of total energy and macronutrient intake.

### 3.4. Biochemical variables

Table 7 presents the pre- and post-intervention values of biochemical markers for the total sample (ALL), the placebo group (PLA), and the experimental group (EXP). No statistically significant within-group pre-post changes (*p* > 0.05) were observed in any biochemical variable in either the PLA or EXP groups when analysed separately (Table 7). However, between-group analyses of change scores using a Generalised Linear Model revealed significant differences for LDH (*p* = 0.04) and CRP (*p* = 0.02), with a trend toward significance for CK (*p* = 0.062) (Figure 2). On the other hand, some trends and effect sizes are worth noting. In the PLA group, the largest mean increase was observed in CK (Δ = 112.92 ± 94.37 UI/L; ES = 1.5), although the change was not statistically significant (*p* = 0.814). CRP also showed a moderate increase (Δ = 0.03 ± 0.03 mg/dL; ES = 1.43), suggesting a potential inflammatory response. The average effect size in this group was moderate (mean ES = 0.89).

In contrast, the EXP group showed a decrease in LDH (Δ = −55.58 ± 51.54 UI/L; ES = 1.5) and CK (Δ = −32.67 ± 47.03 UI/L; ES = 0.74), with large effect sizes despite non-significant *p*-values. These reductions may suggest a potentially favourable biomarker response to CPH supplementation; however, the absence of statistical significance precludes definitive conclusions regarding efficacy.

Figure 2 illustrates the between-group comparison of post-intervention changes. Glucose levels increased slightly in all groups, with the EXP group showing the highest mean change (+2.75 mg/dL; +3.21%). LDH levels decreased in both the ALL and EXP groups (−7.13% and −15.93%, respectively), while a modest increase was observed in the PLA group (+2.31%). Creatine kinase (CK) showed a marked increase in PLA (+112.92 UI/L; +39.99%), whereas it decreased in EXP (−32.67 UI/L; −14.83%), suggesting a potential protective effect of the intervention. Myoglobin levels rose in PLA (+11.75%) but declined in EXP (−7.34%), further supporting a possible attenuation of muscle damage in the experimental group. Insulin levels increased across all groups, with the EXP group exhibiting the greatest relative change (+16.80%). C-reactive protein (CRP) increased in PLA (+24.29%) and ALL (+9.27%), while it decreased in EXP (−8.40%), indicating a potential anti-inflammatory effect of the intervention.

**Table 7.** Within-group pre-post comparisons of biochemical markers in the total sample (ALL), placebo group (PLA), and experimental group (EXP).

	PRE-TEST		POST-TEST		DIFFERENCES				
	M ± SD	CI (95%)	M ± SD	CI (95%)	M ± SD	CI (95%)	**p* value*	Effect Size	
	**ALL**								
Glucose (mg/dl)	86.42 ± 6.29	83.76–89.07	88.58 ± 7.72	85.32–91.84	2.17 ± 6.71	−0.67–5.00	0.115	0.31	
LDH (UI/L)	337.00 ± 125.50	284.00–390.00	312.96 ± 50.80	291.51–334.41	−24.04 ± 133.55	−80.43–32.35	0.710	−0.25	
CK (UI/L)	251.29 ± 156.27	185.30–317.28	291.42 ± 268.03	178.24–404.60	40.13 ± 263.32	−71.07–151.32	0.855	0.18	
Myoglobin (ng/ml)	53.08 ± 25.40	42.36–63.81	54.33 ± 21.78	45.14–63.53	1.25 ± 23.93	−8.85–11.35	0.511	0.05	
Insulin (mcU/mL)	3.55 ± 1.71	2.82–4.27	3.98 ± 1.56	3.32–4.64	0.44 ± 1.14	−0.04–0.92	0.067	0.27	
CRP (mg/dl)	0.11 ± 0.05	0.09–0.13	0.12 ± 0.06	0.09–0.14	0.01 ± 0.08	−0.02–0.04	0.398	0.18	
	**PLA**								
Glucose (mg/dl)	87.25 ± 1.58	83.77–90.73	88.83 ± 1.96	84.53–93.14	1.58 ± 1.60	−1.94–5.11	0.266	0.89	
LDH (UI/L)	325.08 ± 10.51	301.94–348.22	332.58 ± 15.62	298.21–366.95	7.50 ± 16.44	−28.69–43.69	0.666	0.56	
CK (UI/L)	282.33 ± 34.29	206.87–357.80	395.25 ± 98.06	179.43–611.07	112.92 ± 94.37	−94.79–320.63	0.814	1.54	
Myoglobin (ng/ml)	53.92 ± 7.08	38.34–69.50	60.25 ± 8.10	42.43–78.07	6.33 ± 4.79	−4.22–16.88	0.272	0.83	
Insulin (mcU/mL)	4.02 ± 0.57	2.77–5.27	4.38 ± 0.50	3.28–5.47	0.36 ± 0.33	−0.36–1.08	0.239	0.67	
CRP (mg/dl)	0.12 ± 0.02	0.07–0.16	0.15 ± 0.02	0.10–0.19	0.03 ± 0.03	−0.04–0.10	0.350	1.43	
	**EXP**								
Glucose (mg/dl)	85.58 ± 2.07	81.04–90.13	88.33 ± 2.56	82.70–93.97	2.75 ± 2.28	−2.28–7.78	0.264	1.18	
LDH (UI/L)	348.92 ± 51.07	236.51–461.32	293.33 ± 11.65	267.68–318.98	−55.58 ± 51.54	−169.02–57.86	0.666	−1.50	
CK (UI/L)	220.25 ± 53.89	101.63–338.87	187.58 ± 30.70	120.02–255.15	−32.67 ± 47.03	−136.19–70.85	0.790	−0.74	
Myoglobin (ng/ml)	52.25 ± 7.88	34.90–69.60	48.42 ± 3.28	41.21–55.63	−3.83 ± 8.49	−22.52–14.86	0.937	−0.63	
Insulin (mcU/mL)	3.08 ± 0.38	2.24–3.91	3.59 ± 0.39	2.74–4.44	0.52 ± 0.34	−0.24–1.27	0.136	1.35	
CRP (mg/dl)	0.10 ± 0.009	0.08–0.12	0.09 ± 0.008	0.09–0.09	−0.01 ± 0.01	−0.03–0.01	0.317	−0.97	

**Figure 2.**
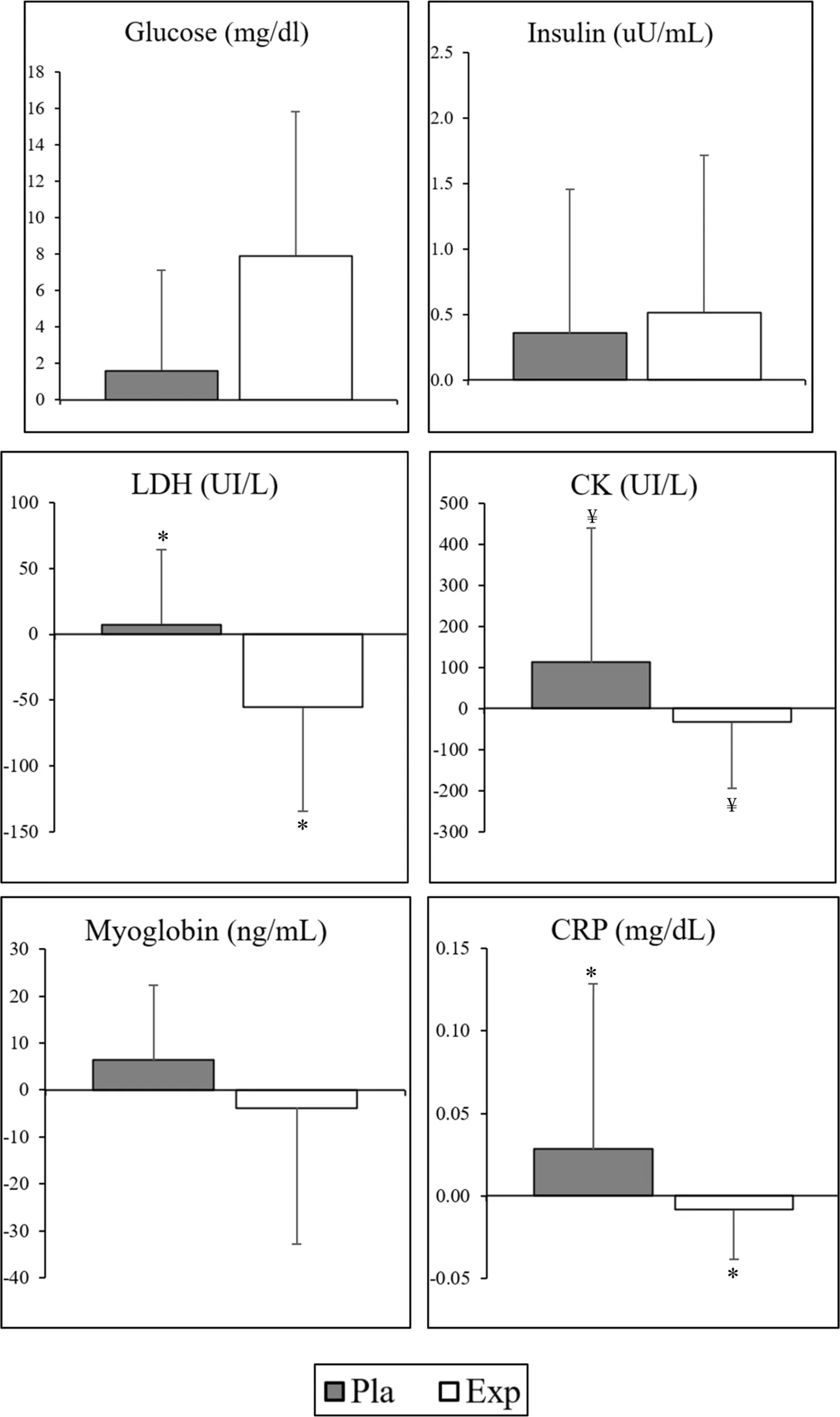
Between-group comparison of post-intervention differences (posttest—pretest). (*) *p* < 0.05, Generalised Linear Model; (¥) *p* = 0.062, Generalised Linear Model.

## 4. Discussion

The aim of the present study was to analyse the effects of CPH supplementation on biomarkers of muscle damage and inflammation and body composition in male road cyclists during a 14-day intervention. Although no significant differences were found in body composition, the biochemical analysis revealed physiologically meaningful trends and significant changes in markers of muscle damage and inflammation. Specifically, the experimental group receiving CPH supplementation demonstrated a significant reduction in LDH and CRP levels, along with a non-significant but moderate effect size reduction in CK, compared with an increase in these biomarkers in the placebo group. These findings suggest that, despite the absence of observable changes in body composition, CPH supplementation may influence selected biomarkers associated with inflammation and muscle damage.

The absence of measurable changes in body composition can be explained by two key factors: the short duration of the intervention and the already high habitual protein intake of the participants. Both groups consumed more than 2 g/kg/day of protein, well above the threshold of ~1.6 g/kg/day identified as sufficient to maximise muscle protein synthesis (MPS) and recovery processes [52,53]. Previous work has shown that, under conditions of high protein availability, the benefits of additional supplementation tend to plateau [54]. Moreover, structural adaptations in lean body mass variables typically require longer interventions—often 6 to 8 weeks or more—before they can be detected [25,27]. Consequently, the lack of significant changes in anthropometrics in this study should not be interpreted as evidence of inefficacy, but rather as a reflection of the study design and population characteristics.

In contrast, the biochemical markers provide valuable insights into the potential of CPH supplementation to influence physiological responses associated with exercise-induced muscle damage and inflammation. The significant between-group difference favoring the EXP group in LDH reduction may suggest a lower degree of exercise-induced cellular stress and membrane disruption [55]. LDH release into circulation is frequently used as an indicator of exercise-induced cellular stress, tissue damage, and oxidative stress [56]. However, LDH is not specific to skeletal muscle, as it is widely distributed across several tissues, including cardiac muscle, liver, and erythrocytes. Therefore, changes in circulating LDH should be interpreted as a general marker of exercise-induced tissue disruption rather than direct evidence of skeletal muscle damage. The reduction observed in the present study may nevertheless suggest a lower degree of exercise-related cellular stress and membrane disruption. This finding aligns with previous evidence showing that hydrolysates from legumes, including chickpeas, can yield bioactive peptides with antioxidant activity capable of neutralising reactive oxygen species [42,44]. Although the physiological magnitude of the observed changes should be interpreted cautiously, the direction of the LDH response is consistent with previous studies conducted with the same hydrolysate, which demonstrated significant antioxidant activity and biological effects on pathways associated with oxidative stress regulation. Therefore, the present findings may provide preliminary in vivo support for mechanisms previously identified under experimental conditions [43,44].

Likewise, the reduction in CRP levels observed in the EXP group, in contrast to the increase in the placebo group, may suggest a modulation of the inflammatory response following exercise. CRP is a sensitive acute-phase protein, and its modulation by plant-derived proteins has been observed in prior studies emphasising anti-inflammatory properties of legume hydrolysates [41,43]. However, these findings should be interpreted with caution. CRP concentrations remained within normal physiological ranges throughout the study in both groups, and the absolute magnitude of the observed changes was small. Consequently, while the between-group differences reached statistical significance, their clinical or practical relevance for trained athletes remains uncertain. These results may therefore be more indicative of subtle changes in the inflammatory response rather than evidence of a meaningful anti-inflammatory effect. Nevertheless, the observed pattern remains biologically plausible considering that the same chickpea protein hydrolysate has previously demonstrated the capacity to modulate inflammatory signalling pathways, including NF-κB and NLRP3-related responses, under controlled experimental conditions [44]. Therefore, although the practical significance of the CRP changes remains uncertain, the findings are mechanistically consistent with previous evidence.

Creatine kinase (CK) and myoglobin, two of the most widely recognised markers of structural muscle damage, also followed a pattern supporting this interpretation. Although the reduction in CK did not reach statistical significance, the moderate effect size and contrasting increases in placebo suggest that CPH supplementation may attenuate muscle fibre disruption. On the other hand, myoglobin, a smaller molecule, peaks shortly after muscle damage, whereas CK peaks 24–48 hours later [57,58]. Given that samples were taken 12 hours post-exercise approximately, the trends observed in both CK and myoglobin are consistent with early modulation of sarcolemmal integrity. This suggests that the benefits of CPH supplementation may be more evident in recovery windows directly relevant to athletes facing consecutive days of competition or training, although more robust data are needed.

A notable feature of the present results was the considerable inter-individual variability observed across several biomarkers, particularly CK and LDH. Such variability is commonly reported in exercise recovery studies and may reflect differences in training status, recovery capacity, habitual dietary intake, genetic factors, and individual physiological responses to exercise. Consequently, the response to supplementation may not be uniform across all athletes, and the present findings should be interpreted considering this biological variability.

These results should also be interpreted in the broader context of the debate over plant versus animal protein sources. Whey protein is widely regarded as the “gold standard” due to its rapid digestibility, high leucine content, and complete amino acid profile, all of which optimise the stimulation of MPS. In contrast, isolated plant proteins such as soy or pea have been criticised for lower digestibility and suboptimal amino acid composition [16,23,24,59]. Recent systematic reviews confirm that while plant proteins are superior to no supplementation, they remain less effective than animal proteins for promoting muscle hypertrophy and strength [17,59,60]. However, these conclusions are nuanced by emerging evidence showing that well-formulated blends of plant proteins (e.g. pea–rice–potato) can elicit anabolic responses comparable to whey, provided that leucine thresholds (~2.5 g/serving) are achieved [25–27]. Importantly, processing techniques such as hydrolysis—applied to CPH supplementation—further enhance digestibility and release bioactive peptides, offering antioxidant and anti-inflammatory benefits that extend beyond anabolic stimulation. In this regard, CPH supplementation may represent a promising plant-based protein strategy with potential bioactive properties. However, the present study was not designed to compare CPH with whey protein, and therefore no conclusions can be drawn regarding their relative efficacy.

The interpretation of the present findings must also take into account modulating factors such as dosage, duration, and population characteristics. The 12 g/day dose of CPH supplementation used here is relatively modest compared to studies that have employed larger amounts (e.g. 50 g/day soy protein in Shenoy et al., 2016) [61] and observed more robust effects [7]. Furthermore, the short 14-day intervention likely limited the capacity to detect changes in body composition. Nevertheless, the significant reductions in LDH and CRP, alongside moderate CK effects, despite a short timeframe and modest dose, may be consistent with the previously described bioactive properties of CPH [43,44].

Equally important, the high baseline protein intake (>2 g/kg/day) in this sample of trained cyclists may have masked any additional anabolic benefit of the supplement [54]. Consequently, the ability to detect treatment effects attributable to additional protein provision may have been substantially reduced. This consideration should be taken into account when interpreting both the positive and null findings of the study. Interestingly, between-group differences in selected biomarkers were observed despite this already high protein intake; however, whether these responses reflect specific bioactive effects of the hydrolysate cannot be determined from the present study design and requires further investigation. Nevertheless, the observed findings support the rationale for further research examining the potential contribution of the hydrolysate’s previously described antioxidant and immunomodulatory properties in athletic populations.

Beyond effects, these findings align with broader considerations of sustainability in nutrition [62]. Plant-based proteins are increasingly promoted not only for health but also for environmental and ethical reasons, with international organisations such as UN Nutrition calling for a transition from animal to plant-based sources [63–65]. Hydrolysates such as chickpea protein represent a valuable step in this direction, offering a sustainable alternative that combines acceptable anabolic potential with unique bioactive properties [66,67]. From a practical perspective, CPH supplementation could serve as a complementary option within athletes’ recovery strategies, particularly when integrated into diets emphasising sustainability without compromising performance.

Several limitations should be considered when interpreting the present findings. The modest sample size limited statistical power, likely contributing to non-significant results for CK despite clinically meaningful trends. Likewise, the short intervention duration restricted the interpretation of the results to acute biochemical responses and prevented extrapolation to longer-term adaptations in body composition or training-induced physiological outcomes. Functional and perceptual measures such as exercise performance, delayed-onset muscle soreness (DOMS), perceived recovery, and fatigue were not assessed, which limits the practical interpretation of the observed biomarker responses. Similarly, direct measures of muscle protein synthesis using isotopic tracer methodologies were not included, preventing definitive conclusions regarding anabolic responses.

Although dietary intake was monitored throughout the intervention and training loads were comparable between groups, other factors known to influence post-exercise recovery and biomarker responses, including sleep quality, psychological stress, hydration status, perceived fatigue, and individual recovery practices, were not directly assessed. Moreover, while blood samples were collected following a standardised 12-hour recovery period after the final high-intensity training session, complete control of all recovery-related factors was not feasible under real-world training conditions. Consequently, these variables may have contributed to the inter-individual variability observed in the biochemical responses.

Another limitation is that nutritional intake was evaluated at the daily level rather than specifically within the immediate post-exercise recovery window. Therefore, potential differences in the timing and composition of post-exercise meals, particularly regarding carbohydrate and protein intake, may have influenced the measured biomarkers and recovery-related physiological responses. In addition, reliance on self-reported dietary intake records may have introduced some degree of reporting bias.

The study design also presents limitations regarding the interpretation of the supplementation effects. First, no protein-matched control group was included, limiting the ability to determine whether the observed responses were attributable to the bioactive properties of the chickpea protein hydrolysate or simply to the additional protein provided by the supplement. Second, only a single supplementation dose (12 g/day) was examined, preventing the evaluation of potential dose-response relationships. Finally, although the experimental supplement was designed to isolate the effect of the hydrolysate, the intervention evaluated a complete formulation rather than the isolated ingredient itself.

Despite these limitations, the present study contributes novel evidence regarding the potential physiological effects of CPH supplementation in trained cyclists. The attenuation of CRP and LDH levels, together with favourable trends observed for CK, should be interpreted cautiously given the modest sample size and the lack of statistical significance for some outcomes. This suggests that CPH supplementation may be particularly relevant for athletes exposed to repeated high-intensity sessions where efficient recovery is critical. A notable strength of the present study is that the intervention was based on a chickpea protein hydrolysate that had previously undergone extensive physicochemical and biological characterisation. Earlier studies demonstrated high digestibility, excellent solubility, a favourable peptide profile characterised by low-molecular-weight peptides, strong antioxidant activity, and immunomodulatory effects in cellular models [43,44]. Consequently, the present intervention was built upon a product with established biological potential rather than a generic plant protein supplement, providing a stronger mechanistic basis for interpreting the observed responses.

Future research should build upon these findings through longer intervention periods (≥8 weeks), larger and more diverse cohorts, and study designs incorporating protein-matched comparator groups, multiple supplementation doses, and direct comparisons with established protein sources such as whey protein. Particular attention should also be given to populations with lower habitual protein intake or greater susceptibility to exercise-induced muscle damage, including vegan athletes and older adults.

In addition, future investigations should incorporate tighter control of post-exercise nutritional intake and recovery-related behaviours, together with mechanistic assessments such as muscle protein synthesis, redox balance, and cytokine responses. The inclusion of functional and perceptual outcomes, including exercise performance, delayed-onset muscle soreness (DOMS), perceived recovery, fatigue, and subsequent training capacity, as well as sex-specific analyses, would provide a more comprehensive understanding of the efficacy and mechanisms of chickpea protein hydrolysate supplementation. Such approaches will help determine whether the biomarker responses observed in the present study translate into meaningful benefits for recovery, adaptation, and athletic performance.

## 5. Conclusions

In conclusion, 14 days of chickpea protein hydrolysate supplementation did not alter body composition in trained cyclists with high habitual protein intakes. However, the intervention was associated with significant between-group differences in selected biomarkers related to exercise-induced inflammation and cellular stress, as well as favourable trends in markers of muscle damage. These findings suggest a potential modulatory effect of chickpea protein hydrolysate on acute physiological responses to exercise, although the practical significance of these changes remains to be established.

When interpreted alongside previous evidence demonstrating antioxidant and immunomodulatory properties of the same hydrolysate, the present results provide preliminary support for its biological activity in athletes. Nevertheless, the short intervention period precludes conclusions regarding long-term adaptations, recovery outcomes, or performance benefits. Further studies with larger samples, longer durations, and direct assessments of recovery and performance are required before definitive conclusions can be drawn.

## Data Availability

In alignment with Research Data Policies promoting open science, the dataset from this study is accessible to interested researchers via the Figshare repository: https://figshare.com/s/a46a8d32ba739611dc96 (provisional private link).

## References

[1] Jäger R , Kerksick CM , Campbell BI , et al. International society of sports nutrition position stand: protein and exercise. J Int Soc Sports Nutr. 2017;14:20. doi: 10.1186/S12970-017-0177-8 PMC5477153 28642676

[2] Hoffman JR , Falvo MJ . Protein – which is best? J Sports Sci Med. 2004;3:118. PMC3905294 24482589

[3] Kraemer WJ , Ratamess NA , Volek JS , et al. The effects of amino acid supplementation on hormonal responses to resistance training overreaching. Metabolism. 2006;55:282–291. doi: 10.1016/j.metabol.2005.08.023 16483870

[4] Hoffman JR , Ratamess NA , Kang J , et al. Effects of protein supplementation on muscular performance and resting hormonal changes in college football players. J Sports Sci Med. 2007;6:85. PMC3778704 24149229

[5] Hulmi JJ , Kovanen V , Selänne H , et al. Acute and long-term effects of resistance exercise with or without protein ingestion on muscle hypertrophy and gene expression. Amino Acids. 2009;37:297–308. doi: 10.1007/S00726-008-0150-6/FIGURES/4 18661258

[6] Cooke MB , Rybalka E , Stathis CG , et al. Whey protein isolate attenuates strength decline after eccentrically-induced muscle damage in healthy individuals. J Int Soc Sports Nutr. 2010;7:30. doi: 10.1186/1550-2783-7-30 PMC2955583 20860817

[7] Govindasamy K , Parpa K , Katanic B , et al. Effect of plant-based proteins on recovery from resistance exercise-induced muscle damage in healthy young Adults—A systematic review. Nutrients. 2025;17:2571. doi: 10.3390/NU17152571/S1 PMC12348865 40806155

[8] Valenzuela PL , Montalvo Z , Mata F , et al. Effects of beef protein supplementation in Male elite triathletes: a randomized, controlled, double-blind, cross-over study. J Am Coll Nutr. 2021;40:53–60. doi: 10.1080/07315724.2020.1727377 32186977

[9] Lin YN , Tseng TT , Knuiman P , et al. Protein supplementation increases adaptations to endurance training: a systematic review and meta-analysis. Clin Nutr. 2021;40:3123–3132. doi: 10.1016/j.clnu.2020.12.012 33358231

[10] Hansen M , Bangsbo J , Jensen J , et al. Effect of whey protein hydrolysate on performance and recovery of top-class orienteering runners. Int J Sport Nutr Exerc Metab. 2015;25:97–109. doi: 10.1123/IJSNEM.2014-0083 25029703

[11] Huang WC , Chang YC , Chen YM , et al. Whey protein improves marathon-induced injury and exercise performance in elite track runners. Int J Med Sci. 2017;14:648–654. doi: 10.7150/IJMS.19584 PMC5562115 28824296

[12] Jonvik KL , Paulussen KJM , Danen SL , et al. Protein supplementation does not augment adaptations to endurance exercise training. Med Sci Sports Exerc. 2019;51:2041–2049. doi: 10.1249/MSS.0000000000002028 PMC6798744 31525168

[13] Dudgeon WD , Kelley EP , Scheett TP . In a single-blind, matched group design: branched-chain amino acid supplementation and resistance training maintains lean body mass during a caloric restricted diet. J Int Soc Sports Nutr. 2016;13:1. doi: 10.1186/S12970-015-0112-9 PMC4700774 26733764

[14] Segovia SA , Vickers MH , Harrison CJ , et al. Maternal high-fat and high-salt diets have differential programming effects on metabolism in adult Male rat offspring. Front Nutr. 2018;5:1. doi: 10.3389/FNUT.2018.00001 PMC5845870 29564328

[15] Australian Institute of Sports (AIS) Australian Sport Commission . 2004. (accessed on 10 October 2025). Supplements. Available online: https://www.ausport.gov.au/ais/nutrition/supplements/group_a#isolated_protein_supplementA

[16] Tang JE , Moore DR , Kujbida GW , et al. Ingestion of whey hydrolysate, casein, or soy protein isolate: effects on mixed muscle protein synthesis at rest and following resistance exercise in young men. J Appl Physiol (1985). 2009;107:987–992. doi: 10.1152/JAPPLPHYSIOL.00076.2009 19589961

[17] Kerksick CM , Jagim A , Hagele A , et al. Plant proteins and exercise: what role can plant proteins have in promoting adaptations to exercise? Nutrients. 2021;13:1962. doi: 10.3390/NU13061962 PMC8230006 34200501

[18] Davies RW , Carson BP , Jakeman PM . The effect of whey protein supplementation on the temporal recovery of muscle function following resistance training: a systematic review and meta-analysis. Nutrients. 2018;10:221. doi: 10.3390/NU10020221 PMC5852797 29462923

[19] Taylor LW , Wilborn C , Roberts MD , et al. Eight weeks of Pre- and postexercise whey protein supplementation increases lean body mass and improves performance in division III collegiate female basketball players. Appl Physiol Nutr Metab. 2016;41:249–254. doi: 10.1139/APNM-2015-0463 26842665

[20] Patel S . Functional food relevance of whey protein: a review of recent findings and scopes ahead. J Funct Foods. 2015;19:308–319. doi: 10.1016/J.JFF.2015.09.040

[21] Gorissen SHM , Crombag JJR , Senden JMG , et al. Protein content and amino acid composition of commercially available plant-based protein isolates. Amino Acids. 2018;50:1685–1695. doi: 10.1007/S00726-018-2640-5 PMC6245118 30167963

[22] Viroli G , Kalmpourtzidou A , Cena H . Exploring benefits and barriers of plant-based diets: health, environmental impact, food accessibility and acceptability. Nutrients. 2023;15:4723. doi: 10.3390/NU15224723 PMC10675717 38004117

[23] Pinckaers PJM , Smeets JSJ , Kouw IWK , et al. Post-prandial muscle protein synthesis rates following the ingestion of pea-derived protein do not differ from ingesting an equivalent amount of milk-derived protein in healthy, young males. Eur J Nutr. 2024;63:893–904. doi: 10.1007/S00394-023-03295-6 PMC10948472 38228945

[24] Pinckaers PJM , Hendriks FK , Hermans WJH , et al. Potato protein ingestion increases muscle protein synthesis rates at rest and during recovery from exercise in humans. Med Sci Sports Exerc. 2022;54:1572–1581. doi: 10.1249/MSS.0000000000002937 PMC9390237 35438672

[25] Joy JM , Lowery RP , Wilson JM , et al. The effects of 8 weeks of whey or rice protein supplementation on body composition and exercise performance. Nutr J. 2013;12:86. doi: 10.1186/1475-2891-12-86 PMC3698202 23782948

[26] Van Der Heijden INO , Monteyne AJ , West SAM , et al. Plant protein blend ingestion stimulates postexercise myofibrillar protein synthesis rates equivalently to whey in resistance-trained adults. Med Sci Sports Exerc. 2024;56:1467–1479. doi: 10.1249/MSS.0000000000003432 PMC11810040 38537270

[27] Moon JM , Ratliff KM , Blumkaitis JC , et al. Effects of daily 24-Gram doses of rice or whey protein on resistance training adaptations in trained males. J Int Soc Sports Nutr. 2020;17:60. doi: 10.1186/S12970-020-00394-1 PMC7706190 33261645

[28] Bessada SMF , Barreira JCM , Oliveira MBPP . Pulses and food security: dietary protein, digestibility, bioactive and functional properties. Trends Food Sci Technol. 2019;93:53–68. doi: 10.1016/J.TIFS.2019.08.022

[29] Hruby A , Jacques PF . Dietary protein and changes in biomarkers of inflammation and oxidative stress in the framingham heart study offspring cohort. Curr Dev Nutr. 2019;3:nzz019. doi: 10.1093/cdn/nzz019 PMC6483052 31037277

[30] Valko M , Leibfritz D , Moncol J , et al. Free radicals and antioxidants in normal physiological functions and human disease. Int J Biochem Cell Biol. 2007;39:44–84. doi: 10.1016/j.biocel.2006.07.001 16978905

[31] Malaguti M , Angeloni C , Hrelia S . Polyphenols in exercise performance and prevention of exercise-induced muscle damage. Oxid Med Cell Longev. 2013;2013:1–9. doi: 10.1155/2013/825928 PMC3742027 23983900

[32] Pingitore A , Lima GPP , Mastorci F , et al. Exercise and oxidative stress: potential effects of antioxidant dietary strategies in sports. Nutrition. 2015;31:916–922. doi: 10.1016/j.nut.2015.02.005 26059364

[33] Draganidis D , Karagounis LG , Athanailidis I , et al. Inflammaging and skeletal muscle: can protein intake make a difference? J Nutr. 2016;146:1940–1952. doi: 10.3945/jn.116.230912 27581584

[34] Higashida K , Kim SH , Higuchi M , et al. Normal adaptations to exercise despite protection against oxidative stress. Am J Physiol Endocrinol Metab. 2011;301:779–784. doi: 10.1152/AJPENDO.00655.2010 PMC3214004 21750271

[35] Hadžović-Džuvo A , Valjevac A , Lepara O , et al. Oxidative stress status in elite athletes engaged in different sport disciplines. Bosn J Basic Med Sci. 2014;14:56–62. doi: 10.17305/BJBMS.2014.2262 PMC4333958 24856375

[36] Chao WX . Health effects of soy protein and isoflavones in humans. J Nutr. 2008;138:1244S–1249S. doi: 10.1093/jn/138.6.1244s 18492864

[37] Zhang T , Zhao T , Zhang Y , et al. Avenanthramide supplementation reduces eccentric exercise-induced inflammation in young men and women. J Int Soc Sports Nutr. 2020;17:41. doi: 10.1186/S12970-020-00368-3 PMC7382060 32711519

[38] Gaamouri N , Zouhal H , Hammami M , et al. Effects of polyphenol (Carob) supplementation on body composition and aerobic capacity in taekwondo athletes. Physiol Behav. 2019;205:22–28. doi: 10.1016/j.physbeh.2019.03.003 30853622

[39] Tachibana N , Yamashita Y , Nagata M , et al. Soy β-Conglycinin improves glucose uptake in skeletal muscle and ameliorates hepatic insulin resistance in goto-kakizaki rats. Nutr Res. 2014;34:160–167. doi: 10.1016/j.nutres.2013.12.001 24461318

[40] Matemu A , Nakamura S , Katayama S . Health benefits of antioxidative peptides derived from legume proteins with a high amino acid score. Antioxidants. 2021;10:316. doi: 10.3390/ANTIOX10020316 PMC7923761 33672537

[41] Ghribi AM , Sila A , Przybylski R , et al. Purification and identification of novel antioxidant peptides from enzymatic hydrolysate of chickpea (Cicer Arietinum L.) protein concentrate. J Funct Foods. 2015;12:516–525. doi: 10.1016/J.JFF.2014.12.011

[42] Faridy JCM , Stephanie CGM , Gabriela MMO , et al. Biological activities of chickpea in human health (Cicer Arietinum L.). A review. Plant Foods Hum Nutr. 2020;75:142–153. doi: 10.1007/S11130-020-00814-2 32239331

[43] Rodríguez-Martín NM , Márquez-López JC , González-Jurado JA , et al. The immunomodulatory potential of chickpea protein hydrolysate via ROS and NO pathways. Biomed Pharmacother. 2025;182:117794. doi: 10.1016/j.biopha.2024.117794 39721324

[44] Rodríguez-Martín NM , Márquez-López JC , Cerrillo I , et al. Production of chickpea protein hydrolysate at laboratory and pilot plant scales: optimization using principal component analysis based on antioxidant activities. Food Chem. 2024;437:137707. doi: 10.1016/J.FOODCHEM.2023.137707 37922804

[45] López-Martínez MI , Miguel M , Garcés-Rimón M . Protein and sport: alternative sources and strategies for bioactive and sustainable sports nutrition. Front Nutr. 2022;9:926043. doi: 10.3389/FNUT.2022.926043/FULL PMC9247391 35782926

[46] Lucia A , Hoyos J , Chicharro JL . Physiology of professional road cycling. Sports Med. 2001;31:325–337. doi: 10.2165/00007256-200131050-00004 11347684

[47] Jeukendrup AE , Craig NP , Hawley JA . The bioenergetics of world class cycling. J Sci Med Sport. 2000;3:414–433. doi: 10.1016/S1440-2440(00)80008-0 11235007

[48] Mielgo-Ayuso J , Fernández-Lázaro D . Nutrition and muscle recovery. Nutrients. 2021;13:1–4. doi: 10.3390/NU13020294 PMC7909540 33498579

[49] Lucía A , Pardo J , Durántez A , et al. Physiological differences between professional and elite road cyclists. Int J Sports Med. 1998;19:342–348. doi: 10.1055/S-2007-971928 9721058

[50] Jeukendrup AE . Periodized nutrition for athletes. Sports Med. 2017;47:51–63. doi: 10.1007/S40279-017-0694-2 PMC5371625 28332115

[51] Tønnessen E , Sandbakk Ø , Sandbakk SB , et al. Training session models in endurance sports: a Norwegian perspective on best practice recommendations. Sports Med. 2024;54:1–19. doi: 10.1007/S40279-024-02067-4 PMC11560996 39012575

[52] López-Moreno M , Kraselnik A . The impact of plant-based proteins on muscle mass and strength performance: a comprehensive review. Curr Nutr Rep. 2025;14:37. doi: 10.1007/S13668-025-00628-1 39982647

[53] Morton RW , Murphy KT , Mckellar SR , et al. A systematic review, meta-analysis and meta-regression of the effect of protein supplementation on resistance training-induced gains in muscle mass and strength in healthy adults. Br J Sports Med. 2018;52:376–384. doi: 10.1136/BJSPORTS-2017-097608 PMC5867436 28698222

[54] Valenzuela PL , Alejo LB , Montalvo-Pérez A , et al. Pre-sleep protein supplementation in professional cyclists during a training camp: a three-arm randomized controlled trial. J Int Soc Sports Nutr. 2023;20:2166366. doi: 10.1080/15502783.2023.2166366 PMC9848340 36686220

[55] Gomez-Cabrera MC , Carretero A , Millan-Domingo F , et al. Redox-related biomarkers in physical exercise. Redox Biol. 2021;42:101956. doi: 10.1016/J.REDOX.2021.101956 PMC8113051 33811000

[56] Callegari GA , Novaes JS , Neto GR , et al. Creatine kinase and lactate dehydrogenase responses after different resistance and aerobic exercise protocols. J Hum Kinet. 2017;58:65–72. doi: 10.1515/HUKIN-2017-0071 PMC5548155 28828078

[57] Pedersen BK , Fischer CP . Physiological roles of muscle-derived Interleukin-6 in response to exercise. Curr Opin Clin Nutr Metab Care. 2007;10:265–271. doi: 10.1097/MCO.0B013E3280EBB5B3 17414493

[58] Febbraio MA , Pedersen BK . Muscle-derived Interleukin-6: mechanisms for activation and possible biological roles. FASEB J. 2002;16:1335–1347. doi: 10.1096/FJ.01-0876REV 12205025

[59] Zhao S , Xu Y , Li J , et al. The effect of plant-based protein ingestion on athletic ability in healthy People—A Bayesian meta-analysis with systematic review of randomized controlled trials. Nutrients. 2024;16:2748. doi: 10.3390/NU16162748/S1 PMC11357476 39203884

[60] Wolfe RR , Church DD , Ferrando AA , et al. Consideration of the role of protein quality in determining dietary protein recommendations. Front Nutr. 2024;11:1389664. doi: 10.3389/FNUT.2024.1389664/BIBTEX PMC11598328 39606577

[61] Shenoy S , Dhawan M , Sandhu JS . Four weeks of supplementation with isolated soy protein attenuates exercise-induced muscle damage and enhances muscle recovery in well trained athletes: a randomized trial. Asian J Sports Med. 2016;7. doi: 10.5812/ASJSM.33528 PMC5098124 27826398

[62] Munialo CD . A review of alternative plant protein sources, their extraction, functional characterisation, application, nutritional value and pinch points to being the solution to sustainable food production. Int J Food Sci Technol. 2023;59:462–472. doi: 10.1111/IJFS.16467

[63] Burlingame B , Moltedo A , Cafiero C . Global protein sustainability and the united nations, through to the 2030 agenda. Front Nutr. 2024;11:1383898. doi: 10.3389/FNUT.2024.1383898/BIBTEX PMC11520426 39474457

[64] European Commission Sustainable Food System . (accessed on 11 October 2025). Available online: https://food.ec.europa.eu/horizontal-topics/farm-fork-strategy/sustainable-food-consumption_en

[65] United Nations THE 17 GOALS . Sustainable Development. (accessed on 11 October 2025). Available online: https://sdgs.un.org/goals

[66] Gundogan R , Tomar GS , Can Karaca A , et al. Chickpea protein: sustainable production, functionality, modification, and applications In: Nadathur S. , Wanasundara J.P. d. , Scanlin L. , editors. Sustainable Protein Sources: Advances for a Healthier Tomorrow. Second Academic Press; 2024. pp. 185–199. ISBN 9780323916523. doi:10.1016/B978-0-323-91652-3.00007-1

[67] Chandran AS , Suri S , Choudhary P . Sustainable plant protein: an up-to-date overview of sources, extraction techniques and utilization. Sustain Food Technol. 2023;1:466–483. doi: 10.1039/D3FB00003F

